# Healthy Food as a New Technology—The Implications of Technological Diffusion and Food Price for Changes in Eating Habits

**DOI:** 10.3389/fnut.2018.00109

**Published:** 2018-11-22

**Authors:** Anne E. Dohmen, D. Raj Raman

**Affiliations:** Department of Agricultural and Biosystems Engineering, Iowa State University, Ames, IA, United States

**Keywords:** diffusion, obesity, health costs, logistic growth, policy

## Abstract

Diet influences health and poor diets drive up healthcare costs for individuals and society as a whole. Multiple governmental programs in the US have aimed to educate citizens about diet choices, resulting in documented successes, as well as, unintended consequences such as increased food waste. Here we examine some of the relationships between healthy diets, food prices, and wealth by drawing parallels between the diffusion of technological innovation and healthy food diets. We introduce a simple modeling framework to estimate the adoption rates of healthy diets based on income and food prices, and describe the implications of the modeling results for the food industry and for government.

## Introduction

In recent decades, obesity rates in the US have increased substantially^1^. Obesity has multiple negative health effects, including type II diabetes, hypertension, cardiovascular disease, and some cancers^2^. In 2012, Cawley and Meyerhoefer (1) estimated the health issues arising for obese persons increase medical costs by $2.7 k per year compared to a non-obese person. Over the entire US population, this leads to an estimated 6–10% of US health expenditures spent on diseases influenced by obesity (2). According to the US Centers for Disease Control and Prevention (CDC), adult obesity rates continue to climb: 39.8% of adults were considered obese in 2015–2016^3^. The term “obesity epidemic” has been used to describe the prevalence of obesity and its negative influence on human health.

A variety of factors are thought to drive the obesity epidemic, including increased caloric intake, reduced physical exercise, women entering the workforce at increasing rates (reducing the time spent preparing healthy meals at home), and consumer preference for convenient—and not necessarily healthy—meal options (2–5). Bleich et al. (2) studied eating habits in developed countries and discovered higher caloric intake is the driving force behind the obesity epidemic. While obesity is prevalent across all income levels in the US, low-income citizens are more likely to be obese than high-income citizens (6). The increased cost of healthy foods may also contribute to unhealthy eating habits.

Americans appear to be increasingly aware of the importance of a healthy, well-balanced diet^4^. Public schools in the US teach content developed by the United States Department of Agriculture (USDA), based on the USDA dietary guidelines, which specify recommended types and quantities of food to eat, and which are reviewed and updated every 5 years^5^. However, education does not always result in changes in behavior, and large parts of the US population continue to have unhealthy eating habits as defined by the USDA guidelines. Multiple factors beyond education influence eating habits, and in this work, we focus on food consumption patterns—specifically on healthy eating habits and the relationships between diet, food prices, and wealth.

## Government programs to measure and encourage healthy eating

The prevalence and cost of obesity has led the US government to take steps to address the epidemic. This includes understanding how Americans eat^6^, promulgating legislation to encourage better food choices^7^, requiring schools to offering healthier foods for breakfast and lunches^8^, and providing nutrition education (7). These measures have had mixed results. The following paragraphs describe what has been done, as well as, research done after implementation to realize their effectiveness.

Quantifying the “healthiness” of a diet can provide insight on the causes of obesity. When the USDA releases their dietary guidelines, the organization tracks Americans' eating habits to monitor how well they are eating based on these recommendations. This comparison produces a number called the Healthy Eating Index (HEI). The USDA is using the HEI to monitor eating habits, and while many Americans are not meeting the required diet, the overall trend since the turn of the century is one of increasing HEI^4^. Although HEI scores increased from 49.1 to 59.0% from 1999 to 2012, only a small majority of Americans are eating as recommended, and this has major impacts on public health and healthcare costs.

In 2012, the USDA took a national household food acquisition and purchase survey to try to understand the characteristics of people at most risk for obesity^6^. Among other results, the study found that SNAP (Supplemental Nutrition Assistance Program, formerly the Food Stamps program) participants had a lower nutritional quality of household food acquisitions, as well has limited household access to healthy food retailers (8). Other studies also show that lower income Americans generally have a poorer-quality diet compared to their wealthier peers. Gu and Tucker (9) looked at the dietary quality trends of children and adolescents from 1999 to 2012, and while the HEI-2010 scores have improved over this time period, participants in the SNAP, National School Lunch Program, and School Breakfast Program have lower dietary quality than non-participants.

Many participators in SNAP do not choose healthy options when buying their own food (10). The 2008 Food and Nutrition Act defines eligible food items which can be purchased with SNAP dollars as any food or food product for home consumption. It does not differentiate between healthy and unhealthy foods, allowing participants to use buy junk food just as easily as healthier options. Perhaps giving SNAP participants incentives to buy healthy food, such as allowing for a percentage increase in benefits when choosing a healthy item or a percentage decrease in benefits when choosing an unhealthy item would encourage participants to change their eating habits (10).

The government is also encouraging Americans to make better dietary choices when going out to eat by showcasing nutrition information. Federal legislation that went into effect in May 2018 requires restaurants and similar food retail establishments that are part of a chain with 20 or more locations to disclose the calories of standard items on their menus. These businesses must also provide other nutritional information such as total fat, saturated and trans fats, cholesterol, etc. upon request^6^. Breck et al. did a study in 2013 to understand the effectiveness of calorie counts on menus, and found that higher-income patrons used the information to make food choices more than patrons with lower incomes (11).

To improve the nutrition of low-income American children, the Healthy Hunger-Free Kids Act was issued in 2010. It worked to change the nutrition standards of food served in schools. In 2012, requirements for this law were put into effect, requiring schools to increase fruit and vegetable offerings, reduce sodium in meals, require the use of whole wheat flour, and offer only non-fat milk, among other stipulations, in an effort to increase the healthfulness of food being eaten by American children^7^. These laws had variable success across the country. In a 2013 study of 10 school districts in California, students were found to be responding positively to the new meals^9^. Parents in the area overwhelmingly supported the new nutrition standards and were pleased their children would be eating better in school^7^. In another study, however, Amin et al. (12) researched fruit and vegetable consumption before and after the implementation of the new standards. Before the law went into effect, students were not required to take fruits and vegetables and consumed more of these items (0.51 cups before the requirement compared to 0.45 cups after). The study showed waste of fruits and vegetables increasing significantly as well—students discarded 0.39 cups of fruits and vegetables after the standards were implemented, compared to 0.25 cup prior to the new law, a 56% increase. Due to the negative reaction to the 2012 standards, the USDA amended the menu planning laws, allowing flexibility to the requirements for whole grain, low sodium items, and non-fat unflavored milk.

Investigators have attempted to understand the scope and effectiveness of governmental programs. McGeary (7) showed that state and federal funding increased from $0.66 million in just seven states in 1992, to $247 million in all 50 states plus the District of Columbia and Puerto Rico in 2006. The results of the study showed that money spent on nutritional education was successfully reducing the prevalence of obesity and overweight adults in the United States. However, the impacts were greater for higher educated and higher income adults, suggesting education programs have less impact on lower income, less educated individuals. Similarly, Frederick et al. (13) reported reduced rates of obesity for adolescents, but with impacts divided according to the teenagers' socioeconomic status: higher income adolescents' obesity rates decreased, while lower income adolescents' obesity rates increased slightly.

## Non-governmental approaches to measuring diet quality

**Figure 1:**
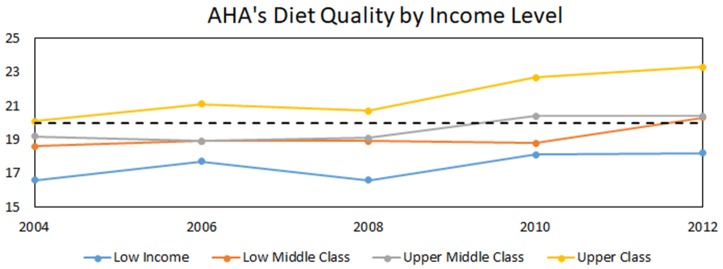
Time course of income-stratified population scores on AHA diets scores. Dotted line represents threshold between poor and intermediate. Adapted from data in Rehm et al. (14).

Another organization focused on eating habits is the American Heart Association (AHA). They have released recommendations for a diet to help reduce cardiovascular disease in America^10^. The AHA diet has a high intake of fruits, vegetables, whole grains, nuts, and fish and tries to minimize sugar, salt, processed meat, and saturated fat. Rehm et al. (14) used data from the National Healthy and Nutrition Examination Survey between 1999 and 2012 to create a point rating system for how healthy a diet is based on the ideal AHA diet. The maximum number of points is 50; poor diets are classified as those meeting <40% (or 20 points) of the diet's goals, while intermediate diets meet 40–80% (or 20–40 points) of the goals. Those meeting the ideal diet of >80% adherence were not included in this analysis. Figure 1 illustrates the stratification of diet quality by income level. Figure 1, based upon (14), depicts that although Americans have improved their diets over the past decade and a half, high-income citizens are improving their diets more rapidly than are those with lower income.

The dotted line represents the watershed between poor to intermediate diet quality in the AHA system. The data shows people of all incomes eating a better diet over time, with higher income people starting at a higher diet quality and increasing diet quality faster than the other brackets.

## Diet choices and food prices

As detailed above, multiple investigations have demonstrated that educational programs seem to have a disproportionate effectiveness for higher income citizens. Understanding how Americans' choose their diet could lead to insight on ways to increase the healthfulness of their diet, especially for low-income citizens. A 2016 study by Beheshti et al. (15) created simulation models which looked at food choices and analyzed these options based on three ways to choosing food; energy cost (price per calorie), unit price (price per gram), and serving price (price per serving). They found dietary food choices for low-income people to be based primarily on price per calorie.

Overlaying this finding—i.e., the importance of price per calorie—with changes in food prices, can provide additional insight into the challenges facing the wider spread adoption of healthy diets. Christian and Rashad (16) looked at changes in the price of food from 1950 to 2007 and found that fruit and vegetable prices increased over time, while the price of snack foods decreased. They were then able to correlate this easier access to calorie dense food to increased obesity rates over time. Fruits and vegetables generally have lower energy densities (lower calories per weight) than foods with refined grains, added sugars and fats. For people with limited incomes, healthier food is difficult to justify.

**Figure 2:**
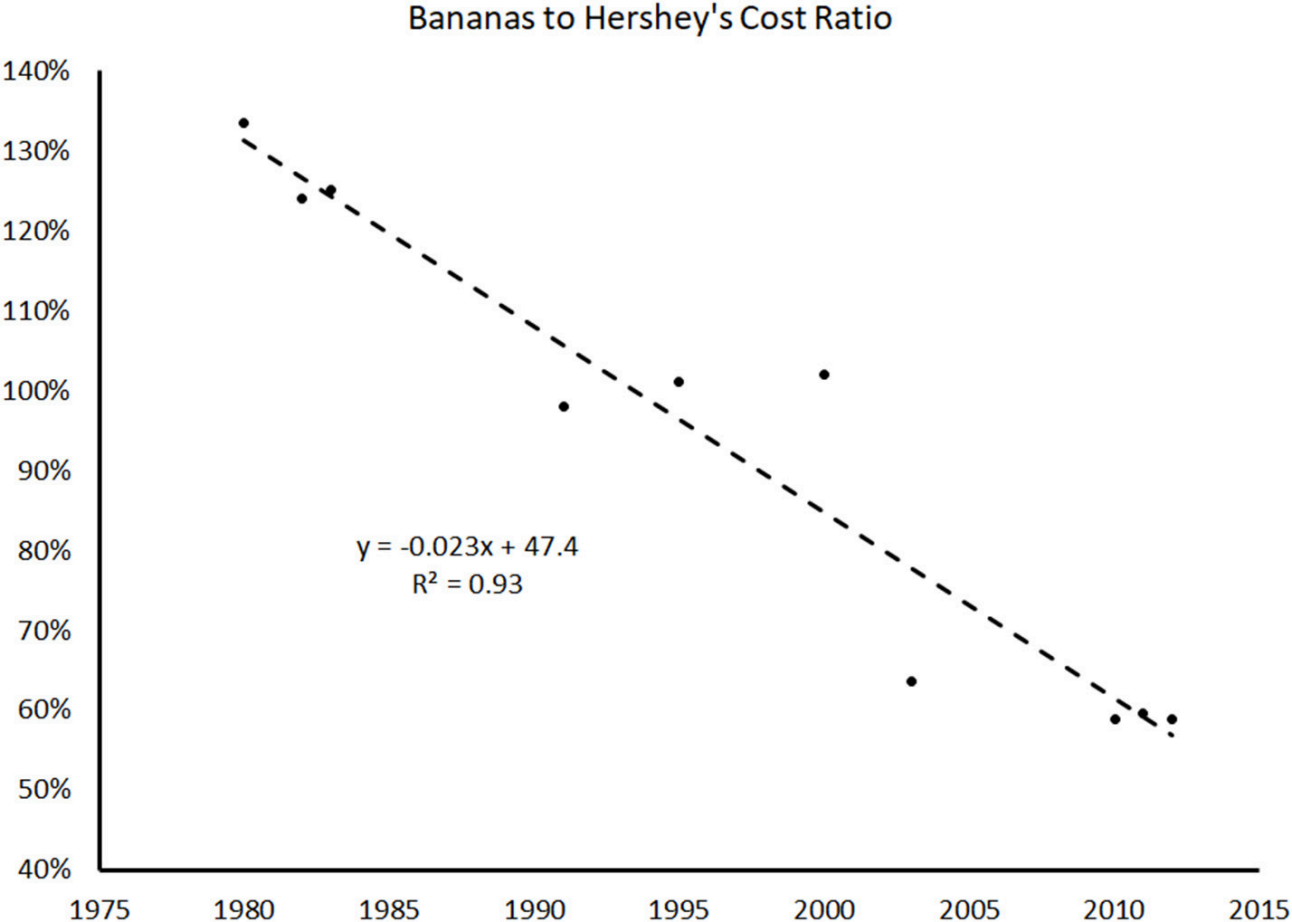
Ratio of exemplar healthy snack cost to that of exemplar unhealthy snack cost, illustrating steady decrease in the relative cost of the two.

We wondered if healthy food prices alone are the critical variable of interest. We decided to explore the *ratio* of certain healthy and unhealthy foods. When we did so, we found that the price ratios of healthy to unhealthy foods was actually decreasing over time. Using historical data (17) of the cost of a banana (an exemplar healthy snack food) and the cost of a representative chocolate candy bar (an exemplar unhealthy snack food) starting in 1980 and ending in 2012 to create a ratio of price for banana to chocolate candy bar creates Figure 2:

Figure 2 shows that in 1980, the cost of a banana was 1.3x the cost of a chocolate candy bar, and in 2012, bananas were only 0.6x the cost of a chocolate candy bar. The best fit linear line shows a 2.3% decrease in the cost ratio of bananas to chocolate candy bars from 1980 to 2012. Even with the limited scope of the data above, knowing that lower income Americans chose food based on price per calorie, the trend of being able to replace a chocolate candy bar with a banana at a similar price could help explain the increase in the HEI over the past few years. The option to adopt a healthier diet is becoming more accessible—the question is how to convince Americans to change the way they eat?

## Healthy food as a “technological innovation”

One explanation for the increase in diet quality could be linked to the knowledge of the effects of obesity. The spread of this awareness could be compared to what Rogers [(18), p. 6] calls the “diffusion of innovations.” In his book of the same name, he states “Diffusion is a kind of social change, defined as the process by which alteration occurs in the structure and function of a social system.” This term can be used both for technology and an idea or practice. Rogers uses an s-shaped curve to show how innovations are adopted in a community. Initially only a few people adopt the new technology. Then as the idea is vetted by the early adopters, use of the new innovation spreads rapidly until it is widely accepted. Finally, adoption slows as it reaches a saturation point, or when the whole population uses the new technology. Examples of diffusion include farming practices, clothing fashions, and internet adoption.

Depending on the innovation, the rate of adoption can vary. Innovations which have a direct and visible advantage are more likely to be accepted faster than those with not so visible or easily understood advantages. Eating well could be considered a preventative innovation, which Rogers describes as an idea that is adopted now to lower the probability of some unwanted event later. These innovations are slow to catch on because the advantages occur in the future, or in this case, may not happen at all; depending on factors such as heredity, thyroid problems, and fitness routines, people can still suffer from diseases associated with obesity even with a healthy diet. An example Rogers uses in his book for a preventative innovation and its rate of adoption is seatbelt use [(18), p. 233]. In 2002, only 73% of Americans used their seatbelts, and 60% of auto deaths were by those not wearing them. When the non-users were asked why they did not use a seatbelt, even if aware of the high risk in the case of an accident, the general consensus was that the cost and effort required to use a seatbelt is greater than the possible benefits. Non-users also felt that the probability of being in an accident was negligible. There are clear parallels between healthy eating and seatbelt use—e.g., persons who have high risk thresholds might not use seatbelts, nor worry about the health of their diet. On the other hand, food has dimensions that reach far beyond calories and health—there are significant socio-cultural aspects to food consumption that make it a more complex realm than the use of safety belts. This is an inherent limitation to our use of this parallel.

**Figure 3:**
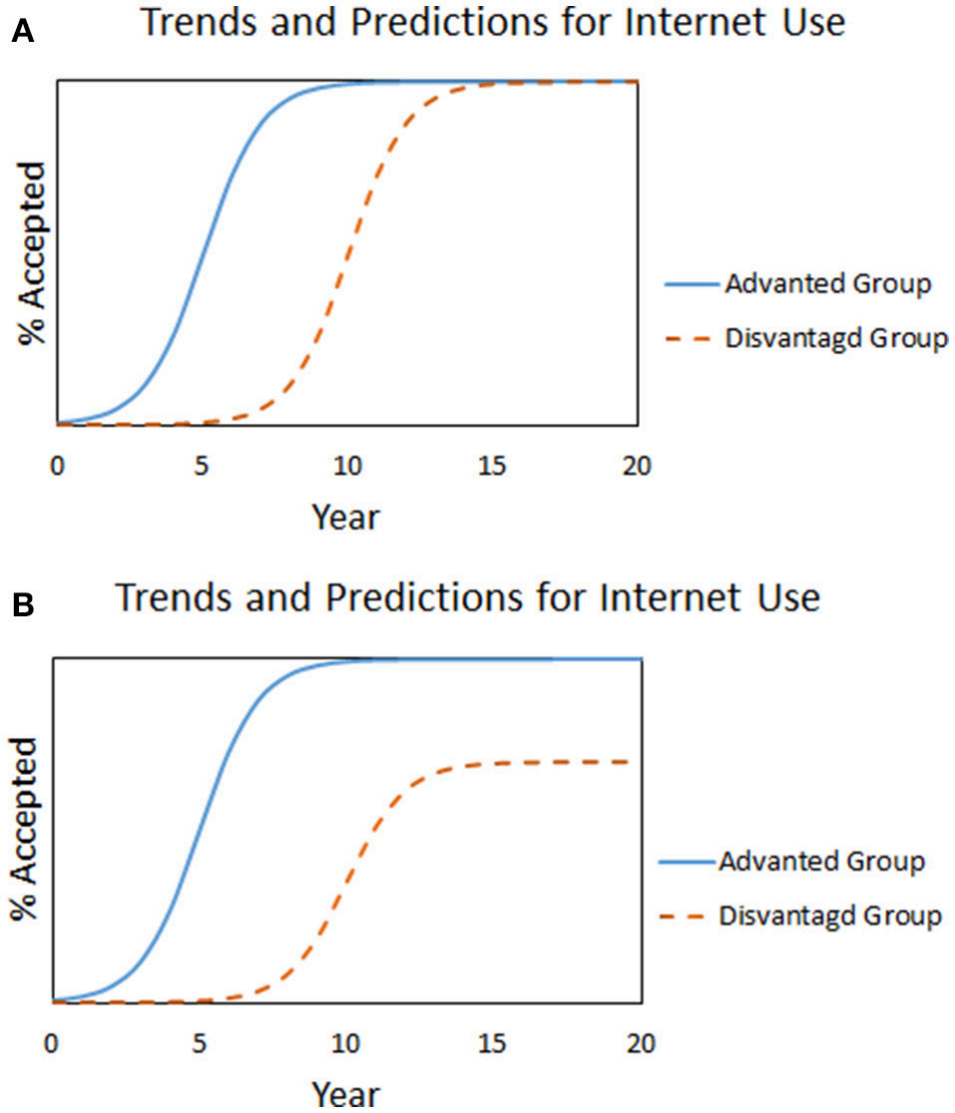
**(A,B**) Internet adoption over time for two wealth-delineated populations, under an assumption of 100% final adoption for both groups **(A)**, and unequal final adoption rates **(B)**. Adapted from Martin and Robinson (19).

In Martin and Robinson (19) used income to predict internet usage in American households. Having access to the internet has been compared to discovery of the alphabet; users who remain without internet face increasing economic, social, health, and other disadvantages. The study aimed to analyze inequality in internet use from 1997 to 2003 by comparing internet adoption to income levels and then predict when these levels would have access to the internet. This study was particularly interesting as income is the variable which most directly correlates with barriers to internet use- the technology had to be seen as useful, as well as, affordable. Their “optimistic” model had everyone eventually getting the internet, but with a dichotomous variable for income- meaning that all groups had the same rate of adoption, but higher income people had a head start on the technology. This follows typical early adopter behavior, and separating adopters based on income instead of lumping everyone together allows for a visual representation of adoption based on economic class. Figure 3A shows both populations reaching 100% internet usage, with economically disadvantaged people reaching full adoption later than advantaged people (poor vs. rich, respectively in this case). In contrast, when the authors assumed that disadvantaged people are unlikely to reach the same final level of adoption, the curve as shown in Figure 3B results.

In the case of healthy eating, graph 3b, which depicts not everyone reaching the same level of eating well, is more likely, especially because it is a preventative innovation.

## Modeling diet choice under assumptions of changing food prices

To do a predictive analysis of eating habits by income using a similar approach as above, we used AHA healthy diet data [from (14)], analyzed as population percentages eating poor, intermediate, or good diets. The researchers found that the vast majority (>95%) of the population was in either the poor diet or intermediate diet category. This makes it possible to simply report the results as percent of population eating the healthier (intermediate) diet verses the poor diet. These results, split between low- and high-income groups (and ignoring the middle-income groups), for a 9-year period, are summarized in the following table:

Two patterns emerge in the table above. The first is that both populations (high- and low-income) are eating better over time—a promising finding, which has been seen in other research. Second, as might be expected if healthy foods command a higher price, thereby making it less accessible to less-wealthy persons, the adoption rates of healthy diets are lower among the low-income population.

To model the adoption rates of an intermediate diet, we used a modification of the Verhulst incarnation of a logistic growth model (20). The unmodified logistic model is as follows:

(1) $$\begin{array}{r} \frac{dP_{i}}{dt}=r_{i}P_{i}\left(1-\frac{P_{i}}{K_{i}}\right) \end{array}$$

Where P_i_ is the fraction of population i adopting a healthy diet (dimensionless), r_i_ is the intrinsic adoption rate for population i (dimensions of inverse time), and K_i_ is the steady-state maximum fraction of population i expected to achieve the intermediate diet (dimensionless).

Our modification to Verhulst's approach involved including the impact of food price on adoption rate by making the variable r_i_ a function of food price, specifically by making it inversely proportional to food price, per the following equation:

(2) $$\begin{array}{r} r_{i}=\frac{b_{i}}{d} \end{array}$$

Where b_i_ is the price insensitivity—different for each income group because people with more disposable income have higher price insensitivity, as they are more likely to adopt healthy foods that cost more—and d is the cost of the technology. The variable d is not subscripted, as the cost is assumed constant across wealth groups. Discretizing the equation (Δt = 1 year), the following equation is used to estimate the growth rate of adoption for healthy foods:

(3) $$\begin{array}{r} P_{i,\;n}=P_{i,\;n-1}+\frac{b_{i}^{*}P_{i,n-1}}{d}*\left(1-\frac{P_{i,n-1}}{K_{i}}\right) \end{array}$$

We used 2017 seat belt adoption rates, which were separated by income bracket, to set the K_i_ values as 90.1% for high-income and 86.7 for low-income populations^11^ Seat belts are a preventative innovation that have been universally available in US passenger vehicles for half a century, and required by law for much of that time. Seat belts are virtually zero-cost because of their ubiquity (and perhaps arguable negative cost due to the fines associated with not wearing them).

Utilizing the starting values from Table 1 to populate P_i_, _2003/4_. We set d to an initial value of 1.0 (arbitrarily, as it is the b/d lumped parameter that drives the model results), and set it to decrease by 2.3%/year based on the analysis of banana vs. chocolate candy bar cost vs. time from above. We then instantiated equation 3 in Excel for a time step of 2 years and varied the values of b_Low−Income_ and b_High−Income_ such that the 2011/2012 values of Pi were in agreement with the tabulated data. Doing this yielded a b-value for high-income group of 2.0, compared to 0.95 for the low-income group, implying high-income citizens are more likely, by approximately a factor of two, to pay for healthy food than are low-income citizens.

**Table 1:** Rates of adoption of intermediate healthy diets by two income-based populations, as reported by Rehm et al. (14).

	**2003–2004 (%)**	**2005–2006 (%)**	**2007–2008 (%)**	**2009–2010 (%)**	**2011–2012 (%)**
P_Low−Income_	31.9	37.4	31.0	39.3	38.4
P_High−Income_	48.5	51.8	51.1	57.9	62.5

**Figure 4:**
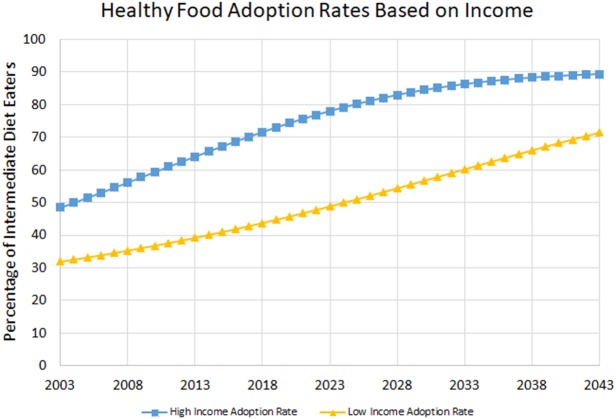
Projected adoption of intermediate health diets by two income groups, based on parameters estimated as described in text.

Although these modeling results must be interpreted with caution, they do allow rough forecasting of the rate at which healthier diets will be adopted by various income groups, as shown in Figure 4 below.

Figure 4 suggests that under a 2.3% annual decrease in the relative price of healthy vs. less-healthy food, nearly 90% of high-income persons will have adopted an intermediate diet in four decades time, while slightly over 70% of low-income persons will have done so. These results are sensitive to the rate of annual decrease of the relative prices of food. If instead of 2.3%, a 3.0% annual decrease is assumed, high-income citizen adoption is roughly the same while low-income citizens achieve 80% adoption in four decades. Conversely, if the rate is only 1.5%, high-income citizens achieve 78% adoption, while low-income achieve only 66% adoption. These results suggest that driving down the cost of healthy foods—both raw and processed—could be a critical approach to improving diets.

This model also only goes through 2012. This is because the data on how Americans are eating based on income has only been analyzed and compiled up to this date. While there likely have been many changes to food prices and eating habits since 2012, the data to extend past this date is not available. It would be interesting to see if current data fits the model above.

## Americans may be opting for healthier processed foods—current food trends

With higher-income Americans eating healthier and demanding a greater selection of healthy products, their purchasing habits at grocery stores and restaurants is changing as well. Kraft, Heinz, Campbell Soup, and J.M. Smucker reported weak sales trends at the end of 2017, and noted that Americans are avoiding once-popular processed food (boxed and canned) in favor of fresher, higher-quality items^12^.

Traditionally, big food manufacturers and fast food restaurants have provided highly accessible unhealthy foods—a trend driven by the desire for profit and the popularity and hence high sales of unhealthy food^13^. Companies are replacing the ingredients in their products with healthier options, and large corporations are purchasing smaller health-food companies in an attempt to leverage the small-company brand and know-how. McDonald's provides a case study: as their sales declined, the company started switching many of their ingredients to healthier alternatives, including 100% real beef, chickens that are not fed human hormones, and using butter instead of margarine^14^,^15^. Another major food player, General Mills Inc. (GMI), started investing in healthier options, most notably with the purchase of Annie's Homegrown in 2014^16^. After this acquisition, GMI became the third largest producer of natural and organic products, with this portfolio predicted to reach $1.5 billion in net sales by 2020^17^.

Grocery stores are also impacted by the changes in consumer preference, and are using their large-volume purchasing power to force suppliers to change their offerings. For example, grocery giant Walmart partnered with an organic products company in 2014, resulting in the offering of many products costing ~25% less than traditional organic products^18^. Though the line was discontinued just 2 years later, Walmart continues to offer organic produce, as well as, their Great Value brand processed foods with organic content^19^. Discount retailer Aldi is also entering the healthy food market by offering organic fruits and vegetables, removing artificial growth hormones from their dairy products, and removing synthetic colors, partially hydrogenated oils, and added MSG from all its private label products, among other health initiatives^20^. Aldi has already disrupted the grocery store market in the UK and is currently investing 3 billion into expanding its US market^21^.

This effort to appeal to high-income Americans is leading to healthier food for lower income Americans as well. Many companies use a platform system in which products are the same regardless of where they are purchased, for example, McDonald's was founded in 1955 with the belief that their food should taste the same in Alaska as it does in Alabama^22^. As larger companies attempt to keep market shares of wealthier consumers (i.e., consumers that have both the desire and means to purchase healthier foods) by altering their products, their use of platform systems implies that all of those new products will be available to lower income consumers as well. In GMI's case, the purchase of Annie's Homegrown has resulted both in a large expansion of available products, and greatly increased availability of those products, which are now distributed widely across the country^23^.

## Conclusion

The obesity epidemic is a complex issue with multiple drivers. But it is not insurmountable, as shown by the success of educational efforts, and by the progression of healthy eating index scores over time. It is important to recognize though that for healthy diet choices to be made by all citizens—not just those with disproportionately high access to resources—factor such as convenience and cost must be addressed. A hopeful trend is the increasing popularity and availability of healthy foods. This trend, when combined with continued educational efforts, has great potential to help larger fractions of the population lead healthier lives.

## Author contributions

AD and DR contributed conception and design of the study. AD collected and organized the articles, wrote the first draft of the manuscript and ran multiple scenarios of the model. DR conceived the approach of considering new-food as technological diffusion, and built the first iteration of the model. Both authors contributed to manuscript revision, read, and approved the submitted version.

### Conflict of interest statement

The authors declare that the research was conducted in the absence of any commercial or financial relationships that could be construed as a potential conflict of interest.

## Footnotes

^1^“Prevalence of Overweight, Obesity, and Extreme Obesity Among Adults Aged 20 and Over: United States, 1960–1962 Through 2013–2014.” Accessed July 25, 2018. https://www.cdc.gov/nchs/data/hestat/obesity_adult_13_14/obesity_adult_13_14.htm

^2^“The Health Effects of Overweight and Obesity|Healthy Weight|CDC,” August 29, 2017. https://www.cdc.gov/healthyweight/effects/index.html

^3^“Adult Obesity Facts|Overweight & Obesity|CDC,” June 12, 2018. https://www.cdc.gov/obesity/data/adult.html

^4^Schap, TusaRebecca E. “The Healthy Eating Index: How Is America Doing? |USDA,” 2016. https://www.usda.gov/media/blog/2016/03/16/healthy-eating-index-how-america-doing

^5^“Highlights, Nutrition Education in Public Elementary and Secondary Schools.” Accessed July 25, 2018. https://nces.ed.gov/surveys/frss/publications/96852/

^6^“USDA ERS - FoodAPS National Household Food Acquisition and Purchase Survey.” Accessed July 25, 2018. https://www.ers.usda.gov/data-products/foodaps-national-household-food-acquisition-and-purchase-survey/

^7^“Labeling & Nutrition - Calorie Labeling on Restaurant Menus and Vending Machines: What You Need To Know.” WebContent. Accessed July 17, 2018. https://www.fda.gov/food/labelingnutrition/ucm436722.htm

^8^“Nutrition Standards for School Meals | Food and Nutrition Service.” Accessed July 17, 2018. https://www.fns.usda.gov/school-meals/nutrition-standards-school-meals

^9^“Atkins Center Study: Students Prefer New, Healthier School Meals.” UC Berkeley School of Public Health, October 16, 2013. http://sph.berkeley.edu/atkins-center-study-students-prefer-new-healthier-school-meals

^10^“The American Heart Association's Diet and Lifestyle Recommendations.” sitecoreprod.heart.org|beta.heart.org|www.heart.org|heart.org|^*^.azurewebsites.net|localhost. Accessed August 6, 2018. https://www.heart.org/en/healthy-living/healthy-eating/eat-smart/nutrition-basics/aha-diet-and-lifestyle-recommendations

^11^“Explore Seat Belt Use in the United States|2017 Annual Report.” America's Health Rankings. Accessed July 23, 2018. https://www.americashealthrankings.org/explore/annual/measure/seatbelt_use/state/ALL

^12^“Big Food Faces Pressure as Consumers Seek Fresh Meals, Snacks - WSJ.” Accessed July 17, 2018. https://www.wsj.com/articles/food-makers-still-searching-for-stronger-u-s-sales-1518791481

^13^“Food for Thought.” The Economist, December 15, 2012. https://www.economist.com/special-report/2012/12/15/food-for-thought

^14^“McDonald's Starts Rollout of Fresh Beef Quarter-Pound Burgers, Cooked Right When Ordered, to U.S. Restaurants.” McDonald's Corporation. Accessed July 17, 2018. /stories/our-food-details/mcdonalds-rolls-out-fresh-beef

^15^“McDonald's Tweaks Recipes: Real Butter Now, Not Margarine, in Its McMuffins.” The Business Times. Accessed July 17, 2018. https://www.businesstimes.com.sg/consumer/mcdonalds-tweaks-recipes-real-butter-now-not-margarine-in-its-mcmuffins

^16^“Financial News Releases.” General Mills InvestorRoom. Accessed July 25, 2018. http://investors.generalmills.com/2014-09-08-General-Mills-To-Acquire-Annies

^17^“General Mills: One of the World's Largest Food Companies.” Accessed July 17, 2018. http://www.generalmills.com/en/News/NewsReleases/Library/2018/March/organicacreage

^18^“Walmart and Wild Oats Launch Effort to Drive Down Organic Food Prices.” Accessed July 17, 2018. https://news.walmart.com/_news_/news-archive/2014/04/10/walmart-and-wild-oats-launch-effort-to-drive-down-organic-food-prices

^19^“Walmart Gets Rid of Organic Wild Oats, Price First Brands|Money.” Accessed July 17, 2018. http://time.com/money/4310142/walmart-brands-whole-foods-aldi/

^20^“ALDI US - Health and Well-Being.” Accessed July 17, 2018. https://corporate.aldi.us/en/corporate-responsibility/customers/health-well-being/

^21^Turner, Zeke. “How Grocery Giant Aldi Plans to Conquer America: Limit Choice.” Wall Street Journal, September 21, 2017, sec. Business. https://www.wsj.com/articles/how-grocery-giant-aldi-plans-to-conquer-america-limit-choice-1506004169

^22^“Our History: Ray Kroc & The McDonald's Brothers|McDonald's.” Accessed July 17, 2018. https://www.mcdonalds.com/us/en-us/about-us/our-history.html

^23^Christenson, Bridget. “Organic Growth on the Rise.” A Taste of General Mills, September 17, 2015. https://blog.generalmills.com/2015/09/organic-growth-on-the-rise/
